# Enhanced recruitment of glutamate receptors underlies excitotoxicity of mitral cells in acute hyperammonemia

**DOI:** 10.3389/fncel.2022.1002671

**Published:** 2022-10-28

**Authors:** Mingxian Li, Zhenqi Liu, Ke Lai, Hanwei Liu, Lina Gong, Haosong Shi, Weitian Zhang, Hui Wang, Haibo Shi

**Affiliations:** Department of Otorhinolaryngology, Shanghai Sixth People’s Hospital Affiliated to Shanghai Jiao Tong University School of Medicine, Shanghai, China

**Keywords:** olfactory dysfunction, ammonium, mitral cell, glutamate receptor, excitotoxicity

## Abstract

Hepatic encephalopathy (HE)–a major complication of liver disease–has been found to increase the risk of olfactory dysfunction, which may be attributed to elevated levels of ammonia/ammonium in the blood and cerebrospinal fluid. However, the cellular mechanisms underlying hyperammonemia-induced olfactory dysfunction remain unclear. By performing patch-clamp recordings of mitral cells (MCs) in the mouse olfactory bulb (OB), we found that 3 mM ammonium (NH_4_^+^) increased the spontaneous firing frequency and attenuated the amplitude, but synaptic blockers could prevent the changes, suggesting the important role of glutamate receptors in NH_4_^+^-induced hyperexcitability of MCs. We also found NH_4_^+^ reduced the currents of voltage-gated K^+^ channel (Kv), which may lead to the attenuation of spontaneous firing amplitude by NH_4_^+^. Further studies demonstrated NH_4_^+^ enhanced the amplitude and integral area of long-lasting spontaneous excitatory post-synaptic currents (sEPSCs) in acute OB slices. This enhancement of excitatory neurotransmission in MCs occurred independently of pre-synaptic glutamate release and re-uptake, and was prevented by the exocytosis inhibitor TAT-NSF700. In addition, an NH_4_^+^-induced increasement in expression of NR1 and GluR1 was detected on cytoplasmic membrane, indicating that increased trafficking of glutamate receptors on membrane surface in MCs is the core mechanism. Moreover, NH_4_^+^-induced enhanced activity of glutamate receptors in acute OB slices caused cell death, which was prevented by antagonizing glutamate receptors or chelating intracellular calcium levels. Our study demonstrates that the enhancement of the activity and recruitment of glutamate receptor directly induces neuronal excitotoxicity, and contributes to the vulnerability of OB to acute hyperammonemia, thus providing a potential pathological mechanism of olfactory defects in patients with hyperammonemia and HE.

## Introduction

Overwhelming evidence indicates that olfactory dysfunction is associated with liver disease (Burch et al., 1978; Deems et al., 1993; Landis et al., 2004; Temmel et al., 2005; Zucco et al., 2006; Heiser et al., 2018), and 27–76% of patients with liver diseases experience olfactory defects, which can severely affect patient food cravings and intake (Deems et al., 1993; Temmel et al., 2005; Heiser et al., 2018). Hepatic encephalopathy (HE) is a major complication of liver dysfunction, and poor olfactory function has been associated with severe cases. Even patients with minimal HE present with compromised thresholds of odor identification (Temmel et al., 2005; Zucco et al., 2006; Heiser et al., 2018). Ammonium (NH_4_^+^)–a proposed major determinants of HE–is toxic and can lead to functional disturbances in the central nervous system by altering neuronal excitability and synaptic transmission (Back et al., 2011; Oja et al., 2017; Jayakumar and Norenberg, 2018).

Ammonium exerts different neuronal effects in different brain regions. It influences the neuronal physiological properties through increasing intracellular calcium levels and altering cytoplasmic pH (Lazarenko et al., 2017). More importantly, NH_4_^+^ can affect glutamatergic and gamma-aminobutyric acid (GABA) neurotransmission and disturb intracellular signal transduction pathways (i.e., the glutamate-NO-cGMP pathway), resulting in impairment of neuronal plasticity, learning ability, memory, or even death in acute and chronic hyperammonemia (Sanchez-Perez and Felipo, 2006; Cabrera-Pastor et al., 2016; Lazarenko et al., 2017; Sancho-Alonso et al., 2022). In the CA1 region of rat hippocampus, acute NH_4_^+^ administration inhibits neuronal long-term potentiation through GABA-enhancing neurosteroids (Izumi et al., 2013). In the chronic hyperammonemia rat, decreased NR2A and NR2B subunit expressions in the hippocampus lead to impaired learning and memory (Yonden et al., 2010). Acute hyperammonemia in mice can impair cortical inhibitory network and trigger seizures by depolarizing neuronal GABA reversal potential (Rangroo Thrane et al., 2013). In rats with chronic hyperammonemia, the amount of GABA transporter GAT-3 is elevated, resulting in the increased extracellular accumulation of GABA leading to motor in-coordination (Cabrera-Pastor et al., 2018). Mitral cells (MCs), are the projecting neurons in the OB and responsible for olfactory encoding and transmission (Wang et al., 2003; Padmanabhan and Urban, 2010; Boyd et al., 2012; Nagayama et al., 2014). Its apical dendrites ramify in the glomerulus, where they receive glutamatergic inputs from the nerves of olfactory sensory neurons (OSNs) and external tufted cells (ETCs). And MCs also receive inhibitory inputs from the interneurons including periglomerular cells (PG) and granule cells (GCs) through dendrodendritic synapses. Despite compelling evidence of the effects of NH_4_^+^ on neurotransmission and neuronal excitability, its pathological mechanism in the olfactory system has never been explored.

In this study, we investigated whether NH_4_^+^ alters the excitability of MCs and explored the potential cellular mechanism of MC excitotoxicity by NH_4_^+^. Our results demonstrated that 3 mM NH_4_^+^ increases the spontaneous firing rates and attenuates the amplitude by potentiating glutamate receptor activity and suppressing Kv currents, leading to MC hyperexcitability. Furthermore, not only have we found that NH_4_^+^ elevates the amplitude and integral area of spontaneous excitatory post-synaptic currents (sEPSCs), but also NH_4_^+^ administration leads to excitotoxicity and cell death through enhancing the activity and membrane trafficking of glutamate receptors, independently of the alteration in glutamate re-uptake and pre-synaptic glutamate release, suggesting elevated activity and expression of glutamate receptors by NH_4_^+^ underlie increased MC excitability and excitotoxicity in the OB.

## Materials and methods

### Ethical approval

All animal procedures were performed in accordance with the guiding principles of the National Institutes of Health Guide for the Care and Use of Laboratory Animals and were approved by the Ethics Committee of the Shanghai Sixth People’s Hospital affiliated to Shanghai Jiao Tong University School of Medicine. All efforts were made to avoid causing unnecessary pain and suffering as possible.

### Slice preparation and solutions

C57BL/6J mice (aged 15–20 days) were anesthetized with isoflurane before decapitation, as previously described (Chen et al., 2016). The brain was excised and immersed in ice-cold oxygenated artificial cerebrospinal fluid (aCSF) containing (in mM): 124 NaCl, 5 KCl, 1.2 KH_2_PO_4_, 2.4 CaCl_2_, 1 MgCl_2_, 24 NaHCO_3_, and 10 glucose, saturated with 95% O_2_ and 5% CO_2_. The forebrain containing the OB was dissected and sectioned into transverse 300 μm-thick slices using a vibratome (VT-1200s, Leica, Nussloch, Germany). The slices were incubated in aCSF with 95% O_2_ and 5% CO_2_ at 37°C for 40 min to recover cell viability and then maintained at room temperature (23–25°C) before patch-clamp recordings.

### Reagents

All chemicals and drugs were purchased from Sigma-Aldrich (St. Louis, MO, USA), unless otherwise indicated. The common reagents used in the experiment were NH_4_Cl, bicuculline (Bic), strychnine (Stry), DL-2-amino-5-phosphonopentanoic acid (APV) and 1,2,3,4-tetrahydro-6-nitro-2,3-dioxo-benzo[f]quinoxaline-7-sulfonamide disodium salt hydrate (NBQX). We prepared different concentrations (1, 3, 5 and 10 mM) of NH_4_Cl solution with an equimolar mass substitute of NaCl. The pH of NH_4_Cl solution in aCSF saturated with 95% O_2_ and 5% CO_2_ was consistent with that of pure aCSF. DL-*threo*-β-benzyloxyaspartic acid (TBOA) and dynasore were purchased from Tocris (Bristol, United Kingdom). TAT-NSF700 was obtained from Anaspec (Fremont, CA, USA). Bic, Stry, APV, NBQX, TBOA, BAPTA-AM, TAT-NSF700, and dynasore were prepared in stock solution at −20°C and later diluted to a final concentration in aCSF before use. The final concentrations of Bic, Stry, APV, NBQX, TBOA, dynasore, TAT-NSF700, and BAPTA-AM were 10, 3, 50, 20, 30, 40, 5, and 40 μM, respectively. During physiological recordings, MCs were exposed to different reagents through a square-tube gravity perfusion system at a speed of 1 mL/min, continuously gassed with 95% O_2_ and 5% CO_2_. All experiments were performed at room temperature.

### Mitral cell identification

The MCs in the OB slices were identified by their location, size, shape, and primary dendrites of their cell bodies. MC cell bodies were located in the MC layer, had a diameter of >20 μm, and their soma structures were characterized by different shapes (triangles, polygons, or ovoids). The soma was readily identified using a CCD camera with a 60 × water immersion objective attached to an upright microscope (Examiner.A1, ZEISS, Gottingen, Germany).

### Cell-attached recordings

Patch pipettes (resistance of 3–5 MΩ) for the soma recordings were fabricated from borosilicate capillary glass (World Precision Instruments, Sarasota, FL, USA) using a vertical pipette puller (PC-10; Narishige, Tokyo, Japan). To record the physiological activity of MCs without causing perturbation of the intracellular homeostasis, spontaneous action potentials were recorded using a loose-patch cell-attached configuration in the current-clamp mode without any current injection. The pipette was only then filled with aCSF. Data were acquired using a MultiClamp 700B (Axon, 5 kHz low-pass-filtered; 1550, sampled at 50 kHz) and pClamp6 software. The gain of the amplifier was set to the highest possible range below saturation to increase the signal to noise ratio and improve data quality.

### Whole-cell voltage-clamp recordings

To investigate the effects of NH_4_^+^ on the excitatory synaptic receptors of MC, we performed voltage-clamp recordings in a whole-cell configuration. The pipette solution used in all whole-cell recordings contained (in mM):130 K-gluconate, 5 KCl, 0.6 ethylene glycol-bis(β-aminoethyl)-N,N,N′,N′-tetraacetic acid (EGTA), 10 4-(2-hydroxyethyl)-1-piperazineethane sulfonic acid (HEPES), 4 MgCl_2_, 3 Na_2_ATP and 0.3 Na_3_GTP and 10 sodium creatine phosphate dibasic tetrahydrate (adjusted to pH 7.3 with KOH). The bath offset potential and electrode capacitance were compensated for before sealing the cell membrane. The series resistance varied from 5 to 15 MΩ among cells and was adjusted by 75–90% to maintain <15 MΩ throughout the recordings. Cells showing changes in series resistance of >15% during recording were omitted from the analysis. We recorded the spontaneous post-synaptic currents (sPSCs) that displayed long-lasting depolarization and continued to record spontaneous excitatory PSCs (sEPSCs) in the presence of Bic and Stry at a holding potential of −60 mV. Bic and Stry were used to suppress inhibitory currents mediated by GABA and glycine. sEPSCs were recorded in the control (Bic + Stry), drug solutions, and wash solution.

To examine the effect of NH_4_^+^ on excitatory inputs from OSNs to MCs, we placed a bipolar stimulation electrode at the OSN nerve stubs in a subset of experiments to stimulate afferent inputs to the recorded cell. Recordings were made 200–400 μm from the stimulation electrode. The threshold for evoked responses, defined as the stimulation threshold, was measured by gradually increasing the intensity until evoked EPSCs (eEPSCs) or burst firings were triggered. The average stimulation threshold was 2.55 ± 0.23 V. The eEPSC represents as “all or none” response (Gire and Schoppa, 2009). To evoke a stable eEPSC, we used a high intensity of stimulation. Single-pulse stimulations were performed with an interval of 20 s over a recording period of 100 s. Paired-pulse stimulation was conducted with an inter-pulse time interval of 2,000 ms, since evoked EPSCs (eEPSCs) displayed a long refractory period and needed more time to recover to baseline. The stimulations were repeated 10 times with an inter-trial interval of 15 s in the control, drug solutions, and wash solution. We further analyzed the paired-pulse ratio (PPR: P2/P1) to represent changes in pre-synaptic release probability.

Furthermore, raw voltage-gated sodium (Nav) and Kv currents were recorded in voltage-clamp mode at a holding potential of –70 mV and were activated by step depolarization from –70 to 40 mV in 10 mV increments, by using test pulses 500 ms in duration.

### Whole-cell current-clamp recordings

We also recorded the spontaneous action potentials at I_0_ in whole-cell current-clamp mode in the aCSF, and drug solutions. Resting membrane potential (RMP) was measured as well. To measure the membrane resistance (R_m_), the evoked depolarized membrane potential was recorded through injecting a current of 10 pA at the holding potential of −60 mV. The rise time constant (τ) was measured by Clampfit 10.6 software. R_m_ was calculated using the equation: τ = R_m_C_m_, where C_m_ is membrane capacitance.

### Membrane surface protein immunoblotting

The fresh mouse OB tissues were incubated in aCSF and 3 mM NH_4_Cl respectively for 1 h and then washed with cold PBS (1×) and lysed in mild protein lysis buffer (Regent A in cell membrane protein extraction kit, Beyotime, Biotechnology, Shanghai, China) and supplemented with protease inhibitor (1×) (phenylmethanesulfonyl fluoride, Beyotime). This was followed by grinding using a glass homogenizer for 80 times, and subsequent centrifugation at 700 × *g* for 10 min. The suspension was collected and centrifuged at 14,000 × *g* for 30 min. As a result, the membrane proteins were pelleted at the bottom of the centrifuge tube. After discarding the supernatant, we added 200 μl Regent B and centrifuged the solution at 14,000 × *g* for 5 min, and collected the membrane protein solution. The solution was boiled in 1 × SDS sample buffer for 5 min and used for western blot. The samples were centrifuged and supernatant protein was separated on 7.5% sodium dodecyl sulfate-polyacrylamide gel electrophoresis (SDS-PAGE) Gel (Invitrogen, Carlsbad, CA, USA). Separated proteins were transferred onto a NC membrane, blocked in 5% non-fat dry milk for at least 1 h at room temperature and subsequently incubated overnight at 4°C on a shaker with primary antibody against rabbit anti-NR1, rabbit anti-GluR1 (purchased from abclonal, Wuhan, China, 1:1000 dilution) and rabbit anti-Na/K-ATPase (Servicebio, Wuhan, China, 1:1000 dilution). After incubation with primary antibodies, the membranes were incubated with goat anti-rabbit horseradish peroxidase conjugated secondary antibodies (abclonal, 1:5000 dilution) for 1 h at room temperature. Na/K-ATPase was chosen as the loading control. Western blots were performed for at least three times and the densitometry analysis was conducted using Image-J Software.

### Assessment of cell vitality with calcein-AM/PI co-staining

To assess the levels of NH_4_^+^-induced neurotoxicity in MCs, we sectioned OB slices to a thickness of 220 μM with a vibratome (Leica) and pre-treated the slices with control, NH_4_Cl, APV + NBQX, APV + NBQX + NH_4_Cl, TAT-NSF700, TAT-NSF700 + NH_4_Cl, BAPTA-AM, or BAPTA-AM + NH_4_Cl solution for 1 h before incubation with 1 μM calcein-AM and 2 μM propidium iodide (PI) (Solarbio, Beijing, China) for 15 min at 37°C. Following three washes with aCSF, the slices were fixed with 4% paraformaldehyde for 1 h and mounted, with a coverslip. Slices were observed by confocal microscopy (LSM-710, ZEISS, Thornwood, NY, USA), and live cells and apoptotic nuclei were counted at 40 × magnification. To evaluate the vitality of projecting neurons, we acquired the regions of interest, including the external plexiform and MC layers. The number of stained cells was measured using Image-J software (National Institutes of Health, Bethesda, MD, USA).

### Statistical analysis

The amplitude, integral area, and frequency of sEPSCs were used to quantify synaptic events. All electrophysiological data were analyzed by Clampfit 10.2 software (Molecular Devices, San Jose, CA, USA). The parameter of minimum allowed duration in the threshold search was 0.001 or 300 ms to detect the peaks of spontaneous firing or EPSCs, respectively. Peaks were detected automatically, but each event was visually inspected to prevent the inclusion of stochastic artifacts. To quantify the features of spontaneous firing, we fitted the inter-event interval of each recording using a Gaussian function. The 3-min duration of the control period was used for quantitative evaluation after the electrophysiological activity of the cells reached stability. All drug treatments lasted for 10 min, and the time window used for analysis was 5 min, after confirming drug activity. The washout time was 6 min, and the last 3-min recording was used for analysis. The average eEPSC amplitude, integral area, and duration under control conditions were normalized to 1.0 for visual presentation and reported as absolute values in the text. Data were analyzed using SPSS 26.0 software (IBM SPSS, Chicago, IL, USA) and are presented as mean ± standard error of the mean (SEM). GraphPad Prism 9 (GraphPad Software, San Diego, CA, USA) and Adobe Illustrator CC (Adobe Systems, San Jose, CA, USA) were used to generate graphics. Three-group comparisons were evaluated using one-way analysis of variance (ANOVA) with a *post hoc* least significant difference (LSD) test to determine intergroup differences. A paired or unpaired Student’s *t*-test was used for two-group comparisons. Statistical significance was set at *p* < 0.05.

## Results

### Mitral cells exhibited typical spontaneous burst firings and long-lasting excitatory depolarized currents

**FIGURE 1:**
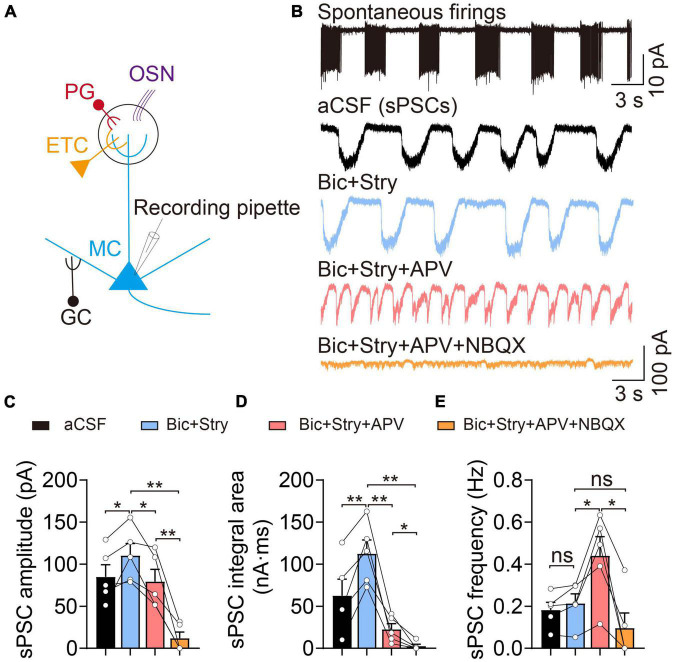
Electrophysiological properties of mitral cells (MCs). **(A)** Basic representation of MC morphology and electrophysiological recording using the patch-clamp technique; olfactory sensory neuron (OSN, purple), external tufted cell (ETC, yellow), periglomerular cell (PG, red), and granule cell (GC, black). **(B)** Cell-attached recording of spontaneous burst firings (upper) and corresponding whole-cell recordings of spontaneous post-synaptic currents (sPSCs) in aCSF, Bic + Stry, Bic + Stry + APV, and Bic + Stry + APV + NBQX in the same MC. **(C–E)** sPSC amplitude, integral area, and frequency in aCSF, Bic + Stry, Bic + Stry + APV, and Bic + Stry + APV + NBQX. Elevated sPSC amplitude and integral area in the presence of Bic + Stry and inhibition of sPSC by APV and NBQX revealed that the excitatory inputs in MC are regulated by glutamate receptors involving NMDA and AMPA receptors. Error bars represent standard error; *p < 0.05, **p < 0.01; ns, not significant; one-way ANOVA with LSD *post-hoc* test.

Mitral cells, as one of the major neurons projecting to the piriform cortex, receive excitatory inputs from OSNs and ETCs, and inhibitory regulation from interneurons, including PG and GCs (Figure 1A). We first explored the electrophysiological properties of MCs in cell-attached voltage-clamp configurations. Previous studies have shown that MCs exert spontaneously active and heterogeneous firing patterns that encode olfactory cues. In our study, most MCs displayed spontaneous and rhythmic burst firing patterns, however other firing characteristics were also present, such as regular and irregular firing (Figure 1B). Interestingly, when the cell membrane was broken through in the voltage-clamp mode at a holding potential of −60 mV, they always appeared waved and long-lasting depolarized currents, defined as sPSCs (also LLD) (Figure 1B). Previous studies have shown the sPSCs consist of both *N*-methyl-D-aspartate (NMDA) and non-NMDA receptor components, and the non-NMDA receptor regulates the initiation of sPSCs (Carlson et al., 2000). The sPSCs are generated by a multistep, diffuse mechanism in the distal portion of the apical dendrite (Schoppa and Westbrook, 2001; Gire et al., 2012). The sPSC amplitude (control: 84.97 ± 14.37 pA, Bic + Stry: 110.20 ± 14.32 pA, *n* = 5, *p* = 0.042) and integral area (control: 62.45 ± 19.66 nA⋅ms, Bic + Stry: 112.40 ± 16.48 nA⋅ms, *n* = 5, *p* = 0.008) were altered by inhibiting GABA_A_ and glycine receptors, while the sPSC frequency remained unchanged in this study (*n* = 5, *p* = 0.150, Figures 1C,D). After applying a cocktail of excitatory and inhibitory synaptic blockers, sEPSC amplitude (Bic + Stry + APV: 79.31 ± 14.54 pA, Bic + Stry + APV + NBQX: 11.96 ± 7.35 pA, *n* = 5), integral area (Bic + Stry + APV: 22.42 ± 6.87 nA⋅ms, Bic + Stry + APV + NBQX: 2.55 ± 2.45 nA⋅ms, *n* = 5), and frequency (Bic + Stry + APV: 0.44 ± 0.09 Hz, Bic + Stry + APV + NBQX: 0.10 ± 0.07 Hz, *n* = 5) were drastically reduced (all *p* < 0.05, Figures 1B–E). Interestingly, the frequency of sEPSCs was significantly increased before and after antagonizing NMDA receptor (Bic + Stry: 0.21 ± 0.04 Hz, Bic + Stry + APV: 0.44 ± 0.09 Hz, *n* = 5, *p* = 0.022). Consistent with previous studies, our results demonstrate that MCs are mainly characterized by spontaneous rhythmic burst firing and long-lasting depolarized currents, which are mainly regulated by glutamate receptors involving NMDA and α-amino-3-hydroxy-5-methyl-4-isoxazole propionate (AMPA) receptors.

### Ammonium enhanced mitral cell excitatory activity by accelerating spontaneous firing rates

**FIGURE 2:**
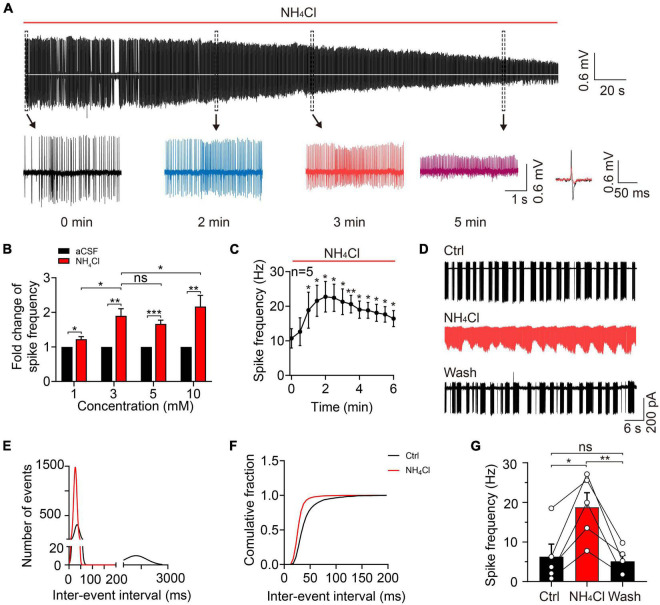
Ammonium enhanced MC excitability by increasing spontaneous firing frequency. **(A)** A typical trace showing spontaneous discharges in a cell-attached recording of a MC in an acute OB slice, showing continuous changes in spike frequency and amplitude with 3 mM NH_4_Cl treatment. Insets below show the traces at 0, 2, 3, and 5 min (expanded from the dashed-line black boxes), as well as the superimposed spike waveforms at 0 (black) and 3 (red) min during NH_4_Cl treatment. **(B)** Fold change in spike frequency in MCs treated with aCSF and NH_4_Cl (1, 3, 5, and 10 mM) in cell-attached recordings. **(C)** Spike frequency of MCs in a cell-attached configuration within 6 min of perfusion with NH_4_Cl solution, showing increased MC excitability. **(D)** An example of spontaneous firing in the cell-attached mode from a MC in control (Ctrl, aCSF solution), 3 mM NH_4_Cl, and washing solution, respectively. **(E)** The relationship between the number of events and inter-event interval of spontaneous firings fitted by Gaussian equation before and after NH_4_Cl application, showing two components in the Ctrl (bin of the first and second component is 5 and 200 ms, respectively) and only one component in NH_4_Cl solution (bin: 5 ms). **(F)** The cumulative fraction of inter-event intervals before and after NH_4_^+^ application, showing much shorter mean intervals between events after NH_4_Cl application. **(G)** The mean frequencies of spontaneous firings before and after NH_4_Cl application, indicating a significant increase in the excitability of MCs by NH_4_Cl. Error bars represent standard error; *p < 0.05, **p < 0.01, ***p < 0.001; ns, not significant; unpaired Student’s *t*-test and one-way ANOVA with LSD *post-hoc* test.

To explore the acute effect of NH_4_^+^ on MC spontaneous firings, we continuously recorded spontaneous action potentials for 10 min with a cell-attached configuration. To determine the appropriate concentration of NH_4_Cl solution in the study, we perfused 1, 3, 5, and 10 mM NH_4_Cl in the OB slices and observed its role in MCs spontaneous firings. We found that 3, 5, and 10 mM NH_4_Cl increased the frequency of spontaneous firings by 89.88% (*n* = 5, *p* = 0.003), 66.42% (*n* = 7, *p* < 0.001), and 116.75% (*n* = 7, *p* = 0.004) over the initial 3-min period, which then weakened as the NH_4_Cl application continued, and the amplitude of the depolarized and hyperpolarized action potential tended to decrease and even disappear (Figures 2A,B). In addition, application of 1 mM NH_4_Cl also increased the spike frequency by 22.39% (*n* = 5, *p* = 0.015) but did not change the amplitude of action potentials. We also observed that high concentrations (e.g., 5 and 10 mM) of NH_4_Cl induced an increment regarding spontaneous firing rates was not much higher than 3 mM NH_4_Cl. Moreover, the percentage of cells with disappearing spikes, were positively associated with the increasing concentrations of NH_4_Cl (0/5 cells in 1 mM NH_4_Cl, 5/10 cells in 3 mM NH_4_Cl, 4/7 cells in 5 mM NH_4_Cl, and 7/7 cells in 10 mM NH_4_Cl). However, these concentrations of NH_4_Cl mentioned above, are higher than the concentration of NH_4_Cl in patients with HE (311 ± 67 μmol/L), and 3 mM NH_4_Cl was an appropriate concentration to explore its acute neuronal excitotoxicity (Ong et al., 2003; Montes-Cortes et al., 2020). Moreover, we found the NH_4_^+^-induced frequency alteration occurred in a time-dependent manner: At first, the frequency was accelerated to approximately three times that of the control and then decreased to a relatively stable frequency with persistent NH_4_Cl application over 6 min at which the frequency was still higher than that in aCSF (*n* = 5, *p* < 0.001) (Figure 2C). Furthermore, to identify whether 3 mM NH_4_Cl could induce a same phenomenon by using HEPES-dialyzed pipette solution, we recorded the spike frequency and RMP in whole-cell current-clamp mode, finding the change of spike frequency is same with cell-attached recordings and a depolarized trend of RMP with a significant transient depolarization within 4-min NH_4_Cl (Supplementary Figure 1).

To clarify the effect of NH_4_^+^ on MC spike activity and whether the NH_4_^+^-induced effect could be reversed, we recorded spontaneous firing in control (aCSF) buffer for 5 min, NH_4_Cl solution for 10 min, and washing solution for 12 min. To analyze the spike frequency, we measured the inter-event interval of 3-min events in the control and NH_4_Cl groups and plotted the data as binned and cumulative frequency histograms (Figures 2D–F). MCs with typical burst firing (in the control) had two components in the fitted curve, the first component representing the inter-event interval of intra-burst firings and the second component showing the inter-burst interval (first component 34.53 ± 10.14 ms, 5-ms bins, second component 1,210 ± 610 ms, 200-ms bins; *n* = 5); following the NH_4_Cl treatment, the curve not only shifted toward zero but transformed into a single component (28.14 ± 6.94 ms, 5-ms bins; *n* = 5; Figure 2E), indicating elevated spike frequency. The left shift of the cumulative curve after NH_4_Cl application indicated a reduction in the mean inter-event interval (Figure 2F). The NH_4_^+^-induced excitation of MCs could be partially reversed by washing solution, but it took ∼10 min for the spike frequency to return to baseline (control: 6.26 ± 3.19 Hz, NH_4_Cl: 18.80 ± 3.65 Hz, Wash: 5.09 ± 1.54 Hz, *n* = 5, *p* = 0.011; Figure 2G). These results demonstrate that NH_4_^+^ can facilitate MC excitation by increasing spontaneous firing frequency, but the mechanisms behind the NH_4_^+^-induced reduction in spike amplitude remain to be explored.

To explore the potential mechanism of NH_4_^+^-induced effects on the frequency and amplitude of spontaneous firings, we conducted the electrophysiological recordings in the presence of APV + NBQX + Bic + Stry. In the experiment, the results showed that NH_4_^+^ temporarily decreased the amplitudes of inward (I_inward_) and outward (I_outward_) currents of spontaneous action potentials and increased the spike frequency within 4-min of NH_4_Cl perfusion which may be due to the depolarized membrane potential initially, but the amplitudes of I_inward_ and I_outward_ and the spike frequency gradually reversed to the baseline (in aCSF) 4-min after which was not consistent with those in the absence of post-synaptic receptors blockers (Supplementary Figures 2A–F). We further detected the effect of NH_4_^+^ on the raw currents of Nav (I_Na_) and Kv (I_k_) channels, showing that the amplitude of I_Na_ was decreased, and the number of I_Na_ events at −40 mV were increased by 4-min NH_4_Cl perfusion, and the changes were recovered at 8-min, while the amplitude of I_k_ were attenuated with 4- and 8-min treatment (Supplementary Figures 3A–D). Interestingly, we also found that the gradual depolarization of RMP by NH_4_^+^ matched with the increased frequency and decreased amplitude of spontaneous firings, indicating NH_4_^+^-induced changes of spontaneous firings may be attributed to the depolarized membrane potential (Supplementary Figures 2G,H). In the experiment, the membrane resistance remained unchanged during NH_4_Cl application (Ctrl: 194.80 ± 18.92 MΩ, NH_4_^+^: 195.30 ± 14.74 MΩ, *n* = 5, *p* = 0.904) (Supplementary Figures 2I,J). In summary, we consider NH_4_^+^-induced changes of spike frequency and amplitude are caused by depolarized membrane potential, but the membrane potential is depolarized transiently when blocking excitatory and inhibitory synaptic inputs, which indicates that overactivity of glutamate receptors but not inhibitory receptors might be the potential factor to aggravate membrane depolarization and evoke MC excitotoxicity because the reversal potential of chloride ion is close to the RMP in the study.

### Ammonium increased the amplitude and integral area of spontaneous excitatory post-synaptic currents

Elevated levels of NH_4_^+^ in the brain can increase glutamine synthesis, which can be catalyzed by glutaminase and decomposed to glutamate. NH_4_^+^ exerts neurotoxicity by facilitating NMDA receptor activation and activating the Ca^2+^-NO-cGMP signaling pathway (Cauli et al., 2009). Spontaneous burst firing was mainly regulated by glutamate receptors in the OB and NH_4_^+^-induced increment of spike frequency could be prevented by synaptic blockers (Hayar et al., 2004; Nagayama et al., 2014). Therefore, we hypothesized that the activity of glutamate receptors was upregulated by NH_4_^+^ resulting in depolarized membrane potential and increased spontaneous MC firing.

**FIGURE 3:**
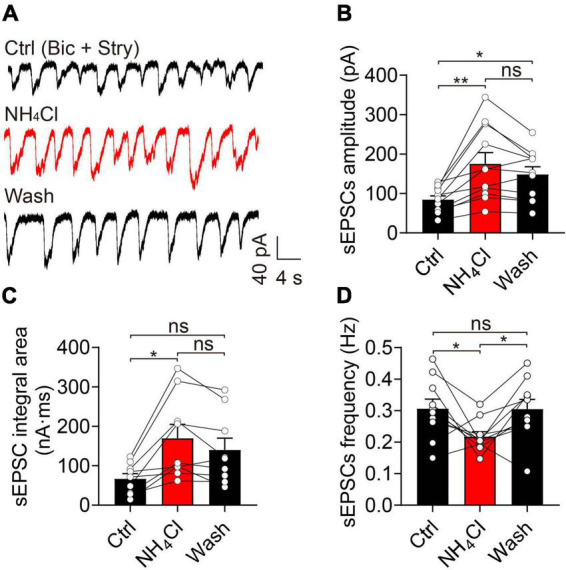
Ammonium (NH_4_^+^) strengthened the excitatory synaptic activity of MCs by increasing sEPSC amplitude and integral area. **(A)** An example of sEPSCs recorded in the voltage-clamp mode in the presence of Bic (10 μM) and Stry (3 μM, Ctrl), indicating increased amplitude and integral area after NH_4_Cl application, which are partially reversed by the wash solution. **(B–D)** sEPSC amplitude, frequency, and integral area in Ctrl, NH_4_Cl, and wash solution, respectively. Error bars represent standard error; *p < 0.05, **p < 0.01; ns, not significant; one-way ANOVA with LSD *post-hoc* test.

NH_4_Cl increased the amplitude of sEPSCs to 168% that of the control, which was partially reversed by washing for 6 min (control: 84.54 ± 9.59 pA, NH_4_Cl: 175.40 ± 28.28 pA, wash: 148.60 ± 19.11 pA, *n* = 11, *p* = 0.012; Figures 3A,B). NH_4_Cl had a similar effect on sEPSC integral area (control: 66.86 ± 12.82 nA⋅ms, NH_4_Cl: 169.40 ± 35.23 nA⋅ms, wash: 139.90 ± 29.95 nA⋅ms, *n* = 9, *p* = 0.042; Figure 3C). In contrast, the sEPSC frequency was reduced from 0.31 ± 0.03 Hz to 0.22 ± 0.02 Hz by NH_4_Cl treatment, and could be reversed to 0.30 ± 0.03 Hz by washing (*n* = 10, *p* = 0.037; Figure 3D). These results indicate that NH_4_^+^ promotes MC neuronal excitability by increasing the amplitude and integral area of sEPSCs.

### Ammonium boosted mitral cell synaptic activity independently of glutamate release from pre-synaptic terminals

**FIGURE 4:**
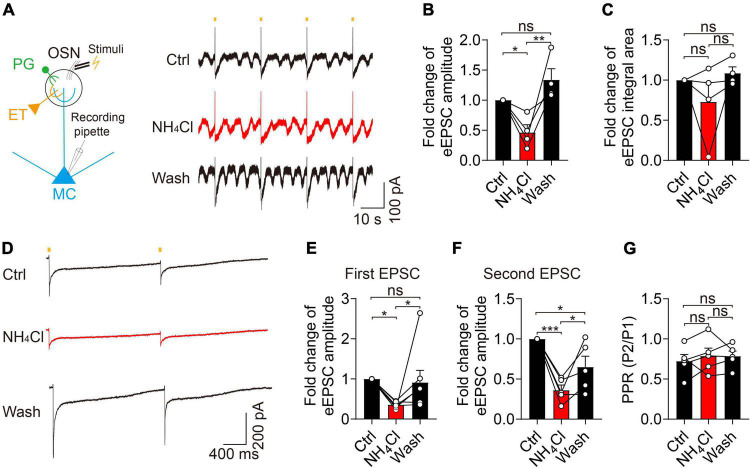
Ammonium (NH_4_^+^) reduced the pre-synaptic glutamate release in MCs without affecting pre-synaptic release probability. **(A)** Left panel showing MC recording of evoked EPSCs (eEPSCs) after stimulating OSN stubs with a bipolar electrode. Right panel showing eEPSCs from a MC by continuous single-pulse stimuli with 20 s intervals in Ctrl, NH_4_Cl, and wash solution, respectively, in the presence of Bic + Stry. **(B–C)** Fold change of eEPSC amplitude, and integral area in Ctrl, NH_4_Cl, and wash solution, respectively. **(D)** An example of eEPSCs recorded from a MC in Ctrl, NH_4_Cl, and wash solution, respectively. EPSCs were evoked by paired-pulse stimuli with an interval of 2,000 ms (determined by the slow recovery course of eEPSCs). **(E–G)** Pooled data showing fold changes of the first and second eEPSC amplitude and PPR (paired-pulse ratio, P2/P1) in Ctrl, NH_4_Cl, and wash solution, respectively. Error bars represent standard error; *p < 0.05, **p < 0.01, ***p < 0.001; ns, not significant; one-way ANOVA with LSD *post-hoc* test.

To determine the potential mechanism of the NH_4_^+^-induced increase of amplitude and integral area of sEPSCs, we recorded eEPSCs by stimulating the olfactory sensory nerves and examined whether NH_4_^+^ could facilitate glutamate release from pre-synaptic terminals. In this experiment, NH_4_^+^ substantially decreased the amplitude of eEPSCs to 46% that of the control during single-pulse stimuli with 20-s intervals (control: 202.20 ± 66.21 pA, NH_4_Cl: 93.61 ± 33.51 pA, wash: 287.30 ± 105.70 pA, *n* = 4, *p* = 0.003; Figures 4A–C), with no change observed in the integral area of eEPSCs (*n* = 4, *p* = 0.250). Washing recovered the amplitude of eEPSCs to an even greater extent than that of the control group (Figure 4B). To test whether the NH_4_^+^-induced reduction in pre-synaptic glutamate release was due to changes in pre-synaptic release probability, we analyzed the PPR (P2/P1). We found that the amplitudes of both the first and second eEPSCs were markedly attenuated by NH_4_Cl (35.79 ± 3.08% that of the control in first EPSCs, *n* = 7, *p* = 0.039; 36.04 ± 6.47% that of the control in second EPSCs, *n* = 5, *p* < 0.001), while the mean PPR was unchanged (*n* = 5, *p* = 0.826; Figures 4D–G). These results suggest that NH_4_^+^ suppresses glutamate release from pre-synaptic terminals without influencing the release probability, which does not explain the enhanced activity of sEPSCs induced by NH_4_^+^. Although we attempted to record miniature EPSCs (mEPSCs) and further confirm the above results, mEPSCs were particularly weak when perfusing 1 μM TTX.

### Blocking glutamate re-uptake did not influence the ammonium-induced increase in excitatory synaptic activity

**FIGURE 5:**
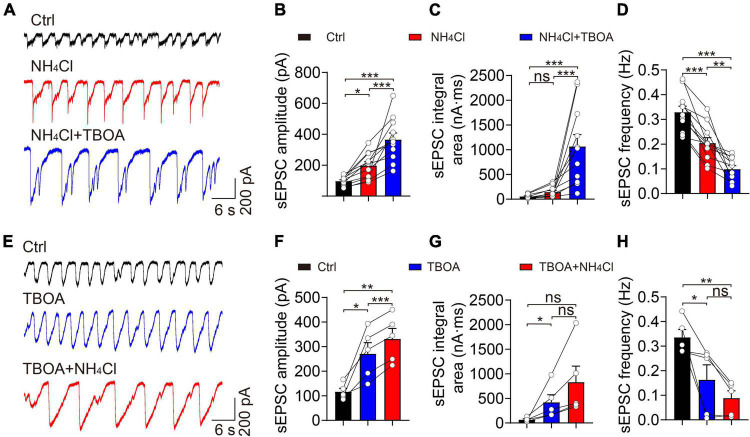
Blockade of EAATs did not attenuate NH_4_^+^-induced potentiation of excitatory synaptic activity. **(A)** Representative traces showing sEPSCs recorded in a voltage-clamp configuration in the presence of Bic + Stry, showing the changes in sEPSC amplitude and integral area in Ctrl, NH_4_Cl, and NH_4_Cl + TBOA (30 μM), respectively. **(B–D)** sEPSC amplitude, integral area, and frequency in sequential treatments with Ctrl, NH_4_Cl, and NH_4_Cl + TBOA. **(E)** Typical sEPSC traces from a MC in sequential treatments of Ctrl, TBOA, and TBOA + NH_4_Cl. **(F–H)** *Post hoc* analysis of sEPSC amplitude, integral area, and frequency in Ctrl, TBOA, and TBOA + NH4Cl, respectively. Error bars represent standard error; *p < 0.05, **p < 0.01, ***p < 0.001; ns, not significant; one-way ANOVA with LSD *post-hoc* test.

Ammonium can inhibit excitatory amino acid transporters (EAATs), which is likely one of the major factors influencing synaptic activity and neuronal excitation in a hyperammonemia model (Butterworth, 1993). Accordingly, we used TBOA to block EAATs in the OB and examined whether NH_4_^+^ could prevent intercellular glutamate re-uptake and, in so doing, promote overactivity of glutamate receptors. We recorded sEPSCs during the sequential application of control, NH_4_Cl, and NH_4_Cl + TBOA solutions (Figure 5A). The TBOA + NH_4_Cl treatment increased the amplitude (control: 96.70 ± 8.64 pA, NH_4_Cl: 196.90 ± 25.10 pA, NH_4_Cl + TBOA: 365.70 ± 42.72 pA, *n* = 11, *p* < 0.001) and integral area (control: 53.75 ± 10.78 nA⋅ms, NH_4_Cl: 142.30 ± 32.52 nA⋅ms, NH_4_Cl + TBOA: 1,067.00 ± 238.10 nA⋅ms, *n* = 11, *p* < 0.001) by 85.73 and 649.82% that of the NH_4_Cl only group, respectively (Figures 5B,C). In contrast, the sEPSCs frequency was reduced by TBOA (control: 0.33 ± 0.02 Hz, NH_4_Cl: 0.20 ± 0.02 Hz, NH_4_Cl + TBOA: 0.10 ± 0.01 Hz, *n* = 11, *p* < 0.001; Figure 5D). Moreover, we first blocked EAATs and then applied NH_4_Cl and found that the mean values of amplitude (control: 116.20 ± 14.41 pA, TBOA: 270.90 ± 45.06 pA, TBOA + NH_4_Cl: 331.50 ± 42.41 pA, *n* = 5, *p* = 0.004) and integral area (control: 67.09 ± 17.37 nA⋅ms, TBOA: 420.50 ± 153.70 nA⋅ms, TBOA + NH_4_Cl: 830.80 ± 325.60 nA⋅ms, *n* = 5, *p* = 0.069) of sEPSCs were also markedly increased by NH_4_^+^ though there were no significantly statistical difference of integral area and frequency between only TBOA and TBOA + NH_4_Cl with one way ANOVA analysis (Figures 5E–H). These results revealed that the activity of EAATs in the OB was not inhibited by NH_4_^+^ and that sEPSCs could still be enhanced by NH_4_^+^ when blocking EAATs. In other words, the enhanced excitatory post-synaptic events induced by NH_4_^+^ in MCs was not attributed to the suppression of glutamate uptake.

### Ammonium enhanced the trafficking of glutamate receptors to the cytoplasmic membrane

**FIGURE 6:**
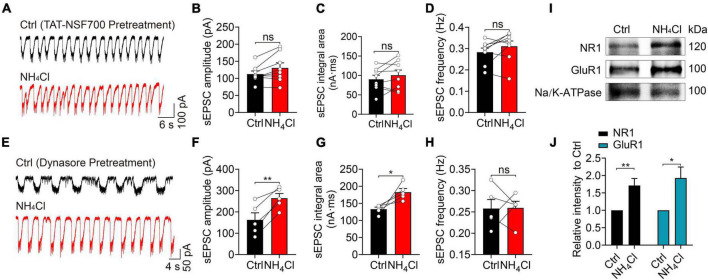
Blocking of exocytosis with TAT-NSF700 or blocking dynamin-dependent endocytosis changes the effect of NH_4_^+^ on the excitability of MCs. **(A)** Example traces of sEPSCs from a MC in the presence of Bic + Stry, showing that the sEPSC amplitude, integral area, and frequency were unchanged by NH_4_Cl after OB slices were pre-treated with TAT-NSF700 (5 μM, 30 min), a permeable thrombin-induced exocytosis inhibitor. **(B–D)** *Post hoc* statistical analysis of sEPSC amplitude, integral area, and frequency in Ctrl and NH_4_Cl solution after OB slices were pre-incubated with TAT-NSF700. **(E)** Typical sEPSC recordings from a MC in Ctrl (Bic + Stry in aCSF) and Ctrl + NH_4_Cl buffer with OB slices pre-incubated with dynasore (40 μM, 30 min), an inhibitor for dynamin-dependent endocytosis. NH_4_Cl effectively augmented sEPSC amplitude and integral area. **(F–H**) Mean sEPSC amplitude, integral area, and frequency in Ctrl (Bic + Stry in aCSF) and NH_4_Cl solution in OB slices pre-incubated with dynasore. **(I)** Representative western blotting images of NR1, GluR1 and Na/K-ATPase from OB in the treatment of aCSF (Ctrl) and NH_4_Cl, respectively. **(J)** Qualification of intensity of NR1 and GluR1 normalized to Ctrl (aCSF). Error bars represent standard error; *p < 0.05, **p < 0.01; ns, not significant; paired Student’s *t*-test, unpaired Student’s *t*-test.

We hypothesized that NH_4_^+^ might increase the expression of glutamate receptors to potentiate excitatory synaptic transmission in MCs. We employed TAT-NSF700, an *N*-ethylmaleimide-sensitive factor (NSF) inhibitor fusion polypeptide that can permeate the cell membrane and interact with intracellular organelles, preventing vesicles from transporting intracellular proteins to the cytoplasmic membrane (Calvert et al., 2007). Pre-treatment of the OB slices with TAT-NSF700 for 30 min effectively alleviated the spontaneous excitatory synaptic activity induced by NH_4_^+^ (Figure 6A); NH_4_^+^ did not change the amplitude, integral area, or frequency of sEPSCs (amplitude: *n* = 8, *p* = 0.052; integral area: *n* = 8, *p* = 0.225; frequency: *n* = 8, *p* = 0.163; Figures 6B–D). This suggests that NH_4_^+^ increased the active recruitment of glutamate receptors from the cytosolic pool to the membrane.

Alternatively, slowed endocytosis of membrane glutamate receptors may also increase sEPSC activity. To explore this, we pre-incubated OB slices with 40 μM dynasore–a dynamin inhibitor that can block the internalization of membrane proteins–and found that NH_4_^+^ still increased the amplitude (control: 162.80 ± 32.85 pA, NH_4_Cl: 264.40 ± 21.91 pA, *n* = 5, *p* = 0.004) and integral area (control: 133.10 ± 5.90 nA⋅ms, NH_4_Cl 182.90 ± 10.46 nA⋅ms, *n* = 5, *p* = 0.033) of sEPSCs by 62.41 and 37.42% that of the control group, respectively (Figures 6E–G). The frequency of sEPSCs was not affected by NH_4_^+^ (*n* = 5, *p* = 0.955) (Figure 6H). Interestingly, blocking the endocytosis of glutamate receptors with dynasore pre-treatment effectively increased NH_4_^+^-induced excitatory synaptic activity (amplitude: NH_4_^+^:175.4 ± 28.28 pA, NH_4_Cl with dynasore: 264.40 ± 21.91 pA, *p* < 0.001; integral area: NH_4_Cl: 169.40 ± 35.23 nA⋅ms, NH_4_Cl with dynasore: 182.90 ± 10.46 nA⋅ms, *p* = 0.039). These results demonstrate that when endocytosis is blocked, NH_4_^+^ can promote the accumulation of glutamate receptors on cell membrane as well as the recruitment of intracellular pool to the cell membrane, thereby synergistically enhancing excitability.

To directly demonstrate whether NH_4_^+^ can increase the recruitment of glutamate receptors to cell membrane surface, we carried out western blotting analysis of glutamate receptor subunits on cell membrane fractions after OB slices were treated with NH_4_Cl solution. Considering that NR1 and GluR1 are the basic subunits of NMDA and AMPA receptor, respectively, we tested the expression of NR1 and GluR1, with Na/K-ATPase as the internal reference. The immunoblotting analysis revealed that NH_4_^+^ significantly elevated the level of NR1 and GluR1 without affecting Na/K-ATPase expression on cytoplasmic membrane (NR1: elevated 1.71 ± 0.20-fold in NH_4_^+^, *n* = 5, *p* = 0.007; GluR1: elevated 1.93 ± 0.32-fold in NH_4_^+^, *n* = 4, *p* = 0.027) (Figures 6I,J). These results further demonstrated that NH_4_^+^ elevates the expression of glutamate receptors, resulting in enhanced MC excitability through promoting the recruitment of glutamate receptors to cytoplasmic membrane.

### Upregulation of glutamate receptors induced by ammonium exacerbated cell death

**FIGURE 7:**
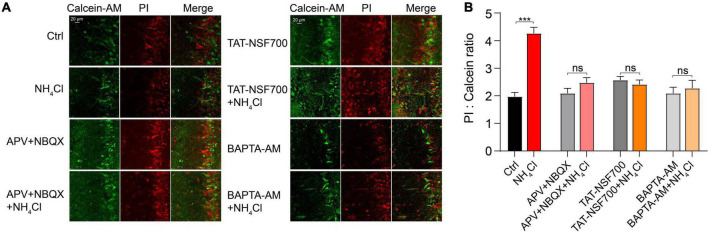
Ammonium (NH_4_^+^)-induced overactivation of glutamate receptors exacerbated MCs death. **(A)** Representative images of live (calcein-AM, Green) and dead cells (PI, Red), and overlay of fluorescence labeling under different experimental conditions with treatment of Ctrl, NH_4_Cl, APV + NBQX, APV + NBQX + NH_4_Cl, TAT-NSF700, TAT-NSF700 + NH_4_Cl, BAPTA-AM, and BAPTA-AM + NH_4_Cl for 1 h. **(B)** Ratio of dead (PI labeling) to live (calcein labeling) cells for all experiments. Error bars represent standard error; ***p < 0.001; ns, not significant; unpaired Student’s *t*-test.

To assess the effect of the upregulated membrane expression of glutamate receptors on cell viability, we assessed the neurotoxicity induced by NH_4_^+^ using a cell death assay. OB slices were pre-treated with control and NH_4_^+^ for 1 h, and then calcein-AM/PI co-staining was applied to quantify the ratio of dead/live cells. NH_4_^+^ increased the dead/live ratio from 1.97 ± 0.16 to 4.26 ± 0.22 that of the control (nine images from three slices, *p* < 0.001), while APV + NBQX, TAT-NSF700, and BAPTA-AM effectively attenuated NH_4_^+^-induced cell death (NH_4_Cl: 4.26 ± 0.22, APV + NBQX + NH_4_Cl: 2.47 ± 0.18, TAT-NSF700 + NH_4_Cl: 2.41 ± 0.16, BAPTA-AM + NH_4_Cl: 2.27 ± 0.29, *p* < 0.001; Figures 7A,B). Interestingly, BAPTA-AM pre-treatment remarkably mitigated the NH_4_^+^-induced cell death (NH_4_Cl: 4.26 ± 0.22, BAPTA-AM + NH_4_Cl: 2.27 ± 0.29, *p* < 0.001, Figures 7A,B). These results indicate that NH_4_^+^ can aggravate cell death, and buffering calcium can suppress the NH_4_^+^-induced neurotoxicity.

## Discussion

Growing evidence demonstrates that patients with liver diseases, especially those with HE, develop a poor olfactory ability to detect and identify odors (Temmel et al., 2005; Zucco et al., 2006). In these patients, serious liver dysfunction can lead to cognitive and motor impairment, coma, and death owing to the accumulation of many metabolic byproducts, among which NH_4_^+^ is a key contributor to cerebral dysfunction (Jayakumar and Norenberg, 2018). The present study provides evidence for NH_4_^+^-induced excitotoxicity of MCs through the enhanced activity of glutamate receptors, revealing the potential mechanism of olfactory impairment in patients with HE.

In the olfactory system, MCs are characterized by burst firing patterns in complex neuronal circuits with neighboring cells (Nagayama et al., 2014). MC has a primary dendrite that forms a highly branched tufts within a single glomerulus, where these tufts receive a multistep signaling input forming glomerulus-specific slow and synchronized bursts or LLD (Gire et al., 2012). These signals include glutamatergic inputs from OSN axons and ETC dendrites, glutamate spillover and lateral excitation between MC-MC/MC-TC dendrodendritic connections, metabolic glutamate receptors, and electrical coupling between dendrites (Nicoll and Jahr, 1982; Isaacson and Strowbridge, 1998; Margrie et al., 2001; De Saint Jan et al., 2009; Gire and Schoppa, 2009; Moran et al., 2021). The initiation of a LLD driven by a glomerulus-specific network, is mainly mediated by AMPA receptors, and NMDA receptors modulate the amplitude and duration of the LLD (Carlson et al., 2000). Our observation that blocking the NMDA receptors results in an increased frequency, which may be attributed to the fast activation and desensitization kinetics of AMPA receptors (Figure 1B).

Interestingly, we also found that 3 mM NH_4_Cl induced significant changes of spontaneous firings in MCs, showing increased frequency and decreased amplitude of spontaneous firings, whereas the increased frequency and decreased amplitude demonstrated to be transient and then recovered after blocking excitatory and inhibitory synaptic inputs. This phenomenon indicates that the enhanced activity of glutamate receptors by NH_4_^+^ plays an essential role in the continuous decrease of spike amplitude, as evidenced by the fact that activation of glutamate receptors in MCs can evoke a long-lasting membrane depolarization (Carlson et al., 2000). Furthermore, Nav and Kv channels are critical for spike initiation, propagation, and firing patterns in central neurons (Catterall et al., 2005; Bean, 2007). Lazarenko et al. has shown NH_4_^+^ depolarized neuronal membrane potential, probably by influencing Na^+^-sensitive background sodium leak channel NALCN (Lu et al., 2007; Lazarenko et al., 2017). NH_4_Cl has been found to be one of the agonists of acid-sensitive ion channels, and may activate the channels contributing to the formation of the transient depolarization of membrane potential (Pidoplichko and Dani, 2006). In our study, transient membrane depolarization and the concurrent suppression of Nav and Kv currents by NH_4_^+^, jointly lead to the amplitude attenuation during the initial NH_4_^+^ application. In addition, previous studies have shown NH_4_^+^ induced the decreased K^+^ conductance and the elevation of extracellular K^+^ which led to membrane depolarization (Allert et al., 1998; Rangroo Thrane et al., 2013). Except for the effect of glutamate receptors, the sustaining attenuation of Kv currents by NH_4_^+^ may be a necessary factor to depolarize membrane potential, resulting in progressive attenuation of amplitude in spontaneous firings.

In our study, we found that acute hyperammonemia can elevate the amplitude and integral area of sEPSCs in MCs, leading to neuronal hyperexcitability and neurotoxicity in OB slices, but the potential mechanism remains to be discussed. At first, olfactory sensory nerves in the superficial OB form monosynaptic connection to the apical dendrite tufts of MCs and the ETCs action potentials mediate the feedforward excitation of MCs in the specific glomerulus (Chen et al., 1997; Hayar et al., 2004; De Saint Jan et al., 2009). In our study, suprathreshold intensity stimulation of OSN stubs did not increase the eEPSC activity in the treatment of NH_4_Cl, indicating the OSN-MC and OSN-ETC-MC synaptic transmission did not play an essential role in NH_4_^+^-induced enhancement of sEPSCs. On the contrary, the pre-synaptic glutamate release was inhibited, consistently with the results of a previous study (Szerb and Butterworth, 1992). Second, it has been shown that ammonia facilitates the synthesis of glutamate and increase the accumulation of extracellular glutamate, which might boost the glutamate spillover and lateral excitation between MC-MC/MC-TC dendrodendritic synapses in the glomeruli (Nicoll and Jahr, 1982; Aroniadou-Anderjaska et al., 1999; Cauli et al., 2009; Cabrera-Pastor et al., 2019). We excluded this possibility because the amplitude and integral area of sEPSCs were still enlarged by NH_4_^+^ after we blocked the uptake of glutamate with TBOA. Third, previous studies have revealed NH_4_^+^ plays a crucial role in triggering neuronal overexcitability by altering glutamate receptor expression in the central nervous system in acute and chronic hyperammonemia (Kosenkov et al., 2018; Sancho-Alonso et al., 2022). However, different results have been reported for glutamate receptor expression in the hippocampus and cerebellum of hyperammonemia rats. Some studies found that chronic hyperammonemia can elevate the membrane expression of NMDA receptors, and alter the expression of AMPA receptor subunits in the hippocampus (Cabrera-Pastor et al., 2016; Hernandez-Rabaza et al., 2016; Kosenkov et al., 2018). However, others found that ammonia reduces MK801 binding to NMDA receptors and the surface expression of the NR1 and NR2A subunits in ammonia-treated cerebellar neurons (Rao et al., 1991; Sanchez-Perez and Felipo, 2006). The C1 domain of NR1 has three serine residues (890, 896, and 897), whose phosphorylation has been implicated in modulating NMDA receptor trafficking and clustering (Llansola et al., 2005). The ammonia-induced increase in phosphorylation of these three serine residues is associated with the expression of NMDA receptors, but the potential mechanism is unclear. In our study, we demonstrated that NH_4_^+^ can dynamically regulate the recycling and trafficking of glutamate receptors and increase their expressions on the membrane in MCs. This process might be due to NH_4_^+^-activated Ca^2+^-dependent post-translational modifications of glutamate receptors on the membrane and/or the recruitment of a pre-existing pool of glutamate receptors.

We acknowledge some limitations to our study. For example, we did not identify the effect of NH_4_^+^ on the neurotransmission of MCs in a glutamate receptor-knockout mouse model. Our study was performed using *in vitro* acute OB slices, which revealed the cellular mechanism of NH_4_^+^ toxicity in the olfactory system; however, it did not consider pathological activity or the influence of NH_4_^+^ on olfactory function in an *in vivo* model.

In conclusion, we investigated the potential mechanisms underlying MC excitotoxicity in an *in vitro* model of acute hyperammonemia. We found that NH_4_^+^ increases spontaneous firing frequency and deceases the amplitude through the joint effects of enhanced glutamate receptor activity and reduced Kv currents. The results also demonstrated enhanced glutamate receptor activity by increasing the recruitment of glutamate receptors on the MC cytoplasmic membrane, contributing to cell overexcitability and neurotoxicity. This study implicates a potential pathological mechanism of olfactory defects in patients with hyperammonemia and HE, and glutamate receptors and their trafficking as potential molecular and cellular targets for protection and intervention against NH_4_^+^-induced neurotoxicity.

## Data availability statement

The original contributions presented in the study are included in the article/Supplementary material, further inquiries can be directed to the corresponding authors.

## Ethics statement

The animal study was reviewed and approved by Ethics Committee of the Shanghai Sixth People’s Hospital Affiliated to Shanghai Jiao Tong University School of Medicine.

## Author contributions

ML, ZL, and KL: experiments and data collection. HBS, HW, and WZ: data analysis and manuscript writing. WZ, HW, HBS, ZL, KL, HL, LG, HSS, and ML: experiment design and manuscript writing. All authors have reviewed the manuscript.

## Supplementary material

Supplementary Figure 1 NH_4_^+^ increased MCs excitability in whole-cell current-clamp mode. **(A)** A recording of spontaneous firings in whole-cell current-clamp mode. **(B,C)** Pooled data showing the time course of resting membrane potential (RMP) and spike frequency during the 6-min application of NH_4_Cl. Error bars represent standard error; **p* < 0.05, ***p* < 0.01, ****p* < 0.001; one-way ANOVA with LSD *post hoc* test.

Supplementary Figure 2 NH_4_^+^ regulated MCs intrinsic excitability transiently. **(A)** Spontaneous firings in the presence of APV + NBQX + Bic + Stry (Ctrl, 1 min) and NH_4_ Cl + APV + NBQX + Bic + Stry(NH_4_Cl, 8 min) in cell-attached mode. **(B)** Spontaneous firings during 0, 2, 4 and 6 min NH_4_Cl application. **(C–E)** Scatter plots showing the amplitudes of inward current (I_inward_), outward current (I_outward_) and the ratio of I_inward_ to I_outward_ in spontaneous action potentials during the time course of NH_4_Cl application. **(F)** Mean frequency during the time course of NH_4_Cl application. **(G)** Whole-cell current-clamp recording of spontaneous action potentials with the injection current of 0 pA in the presence of APV + NBQX + Bic + Stry (Ctrl) and NH_4_Cl + APV + NBQX + Bic + Stry (NH_4_Cl). **(H)** Statistical curve showing the time course of RMP during the application of NH_4_Cl. **(I)** The representative recording when injecting 10 pA current in current-clamp at a membrane potential of −60 mV before (Ctrl) and after NH_4_Cl in the presence of APV + NBQX + Bic + Stry. **(J)** Histogram showing R_*m*_ before (Ctrl) and after NH_4_Cl treatment from **I**, calculated using the function of τ = R_*m*_C_*m*_, where τ is the time constant and C_*m*_ is the membrane capacitance. Error bars represent standard error; **p* < 0.05, ***p* < 0.01, ****p* < 0.001; ns, not significant; one-way ANOVA with LSD post hoc test, paired Student’s *t*-test.

NH_4_^+^-induced changes of voltage-gated sodium and potassium currents. **(A)** Raw electrophysiological traces showing inward currents (Na^+^ current, I_*Na*_) and outward currents (K^+^ current, I_*k*_) in Ctrl, 4-min and 8-min NH_4_Cl. The protocol was performed by injecting step voltages from −70 mV to 40 mV with the increment of 10 mV at the holding potential of −70 mV in the presence of a cocktail of postsynaptic receptors blockers. Right panel representing the expanded I_*Na*_ in left panel. **(B,C)** Voltage-current relationship of I_*Na*_ and I_*k*_ in Ctrl, 4-min and 8-min NH_4_Cl. **(D)** The number of activated I_*Na*_ events when injecting the voltage of −40 mVin Ctrl, 4-min and 8-min NH_4_Cl. Error bars represent standard error; **p* < 0.05, ****p* < 0.001; one-way ANOVA with LSD *post hoc* test.
