# Supplementary figures and images for: Enhanced recruitment of glutamate receptors underlies excitotoxicity of mitral cells in acute hyperammonemia
Source: Front Cell Neurosci. 2022 Oct 28;16:1002671. doi: 10.3389/fncel.2022.1002671 (PMC9651449; doi:10.3389/fncel.2022.1002671)

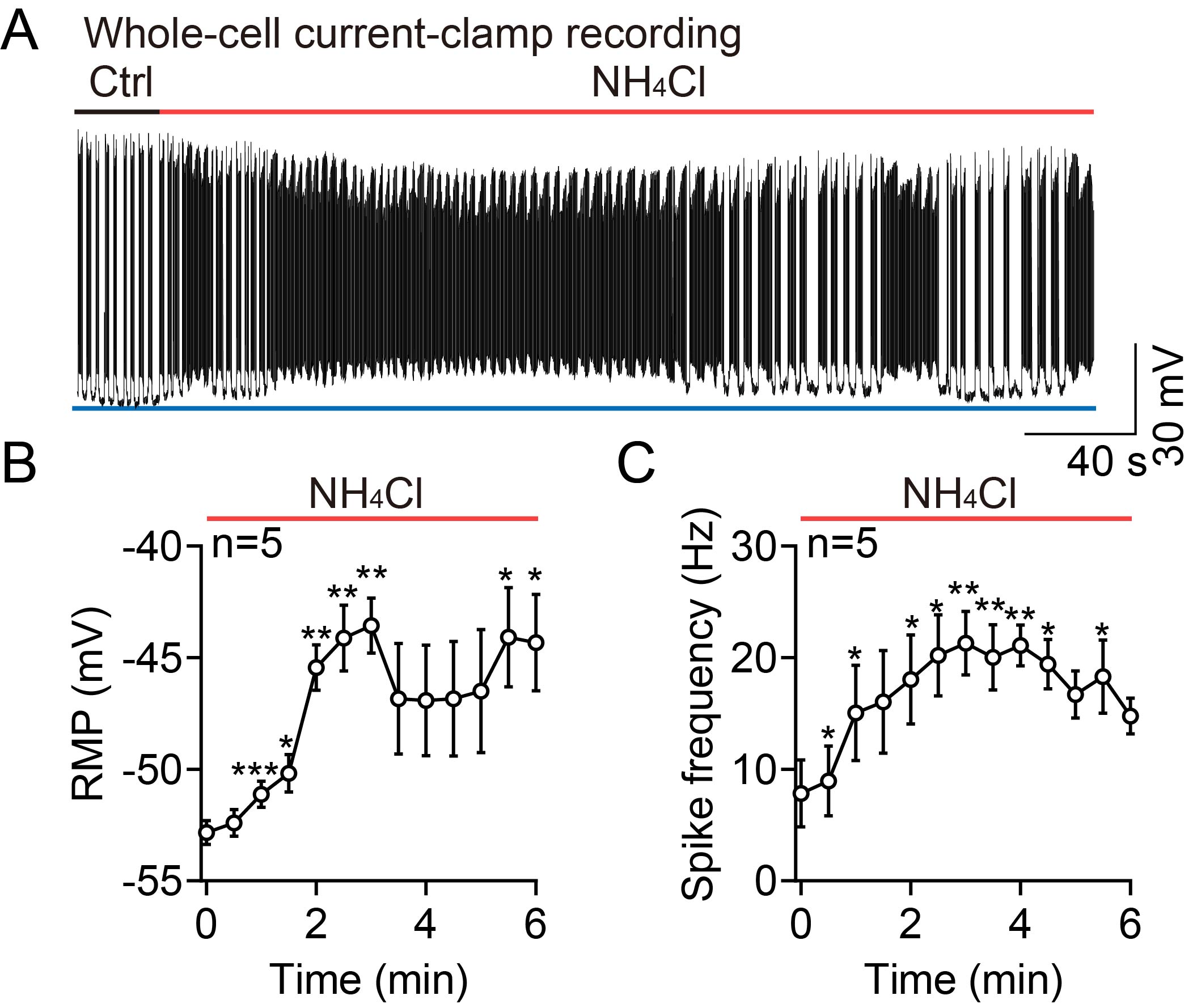

Supplement: Supplementary Figure 1 — NH4+ increased MCs excitability in whole-cell current-clamp mode. (A) A recording of spontaneous firings in whole-cell current-clamp mode. (B,C) Pooled data showing the time course of resting membrane potential (RMP) and spike frequency during the 6-min application of NH4Cl. Error bars represent standard error; *p < 0.05, **p < 0.01, ***p < 0.001; one-way ANOVA with LSD post hoc test. [file Image_1.JPEG]

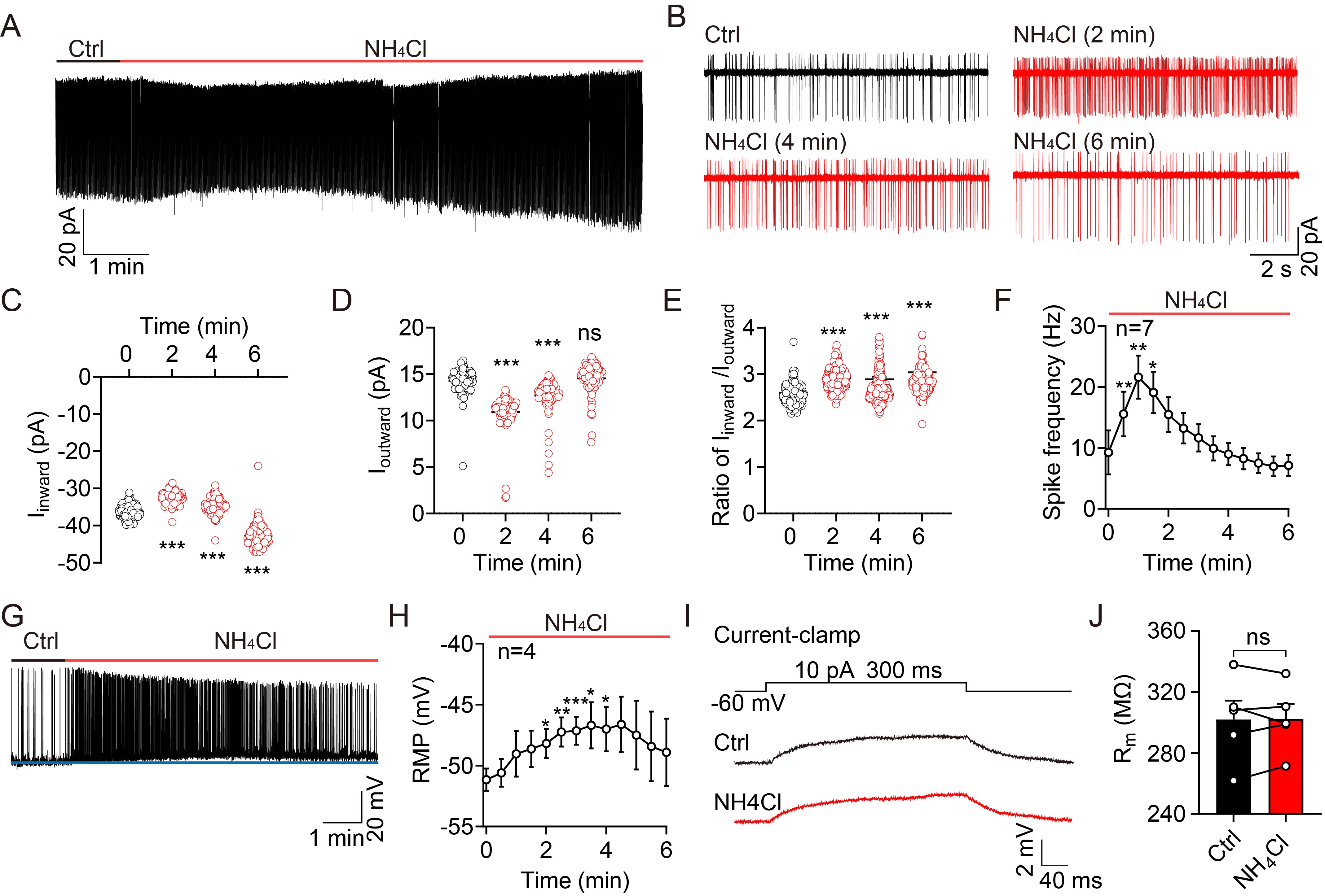

Supplement: Supplementary Figure 2 — NH4+ regulated MCs intrinsic excitability transiently. (A) Spontaneous firings in the presence of APV + NBQX + Bic + Stry (Ctrl, 1 min) and NH4 Cl + APV + NBQX + Bic + Stry(NH4Cl, 8 min) in cell-attached mode. (B) Spontaneous firings during 0, 2, 4 and 6 min NH4Cl application. (C–E) Scatter plots showing the amplitudes of inward current (Iinward), outward current (Ioutward) and the ratio of Iinward to Ioutward in spontaneous action potentials during the time course of NH4Cl application. (F) Mean frequency during the time course of NH4Cl application. (G) Whole-cell current-clamp recording of spontaneous action potentials with the injection current of 0 pA in the presence of APV + NBQX + Bic + Stry (Ctrl) and NH4Cl + APV + NBQX + Bic + Stry (NH4Cl). (H) Statistical curve showing the time course of RMP during the application of NH4Cl. (I) The representative recording when injecting 10 pA current in current-clamp at a membrane potential of −60 mV before (Ctrl) and after NH4Cl in the presence of APV + NBQX + Bic + Stry. (J) Histogram showing Rm before (Ctrl) and after NH4Cl treatment from I, calculated using the function of τ = RmCm, where τ is the time constant and Cm is the membrane capacitance. Error bars represent standard error; *p < 0.05, **p < 0.01, ***p < 0.001; ns, not significant; one-way ANOVA with LSD post hoc test, paired Student’s t-test. [file Image_2.JPEG]

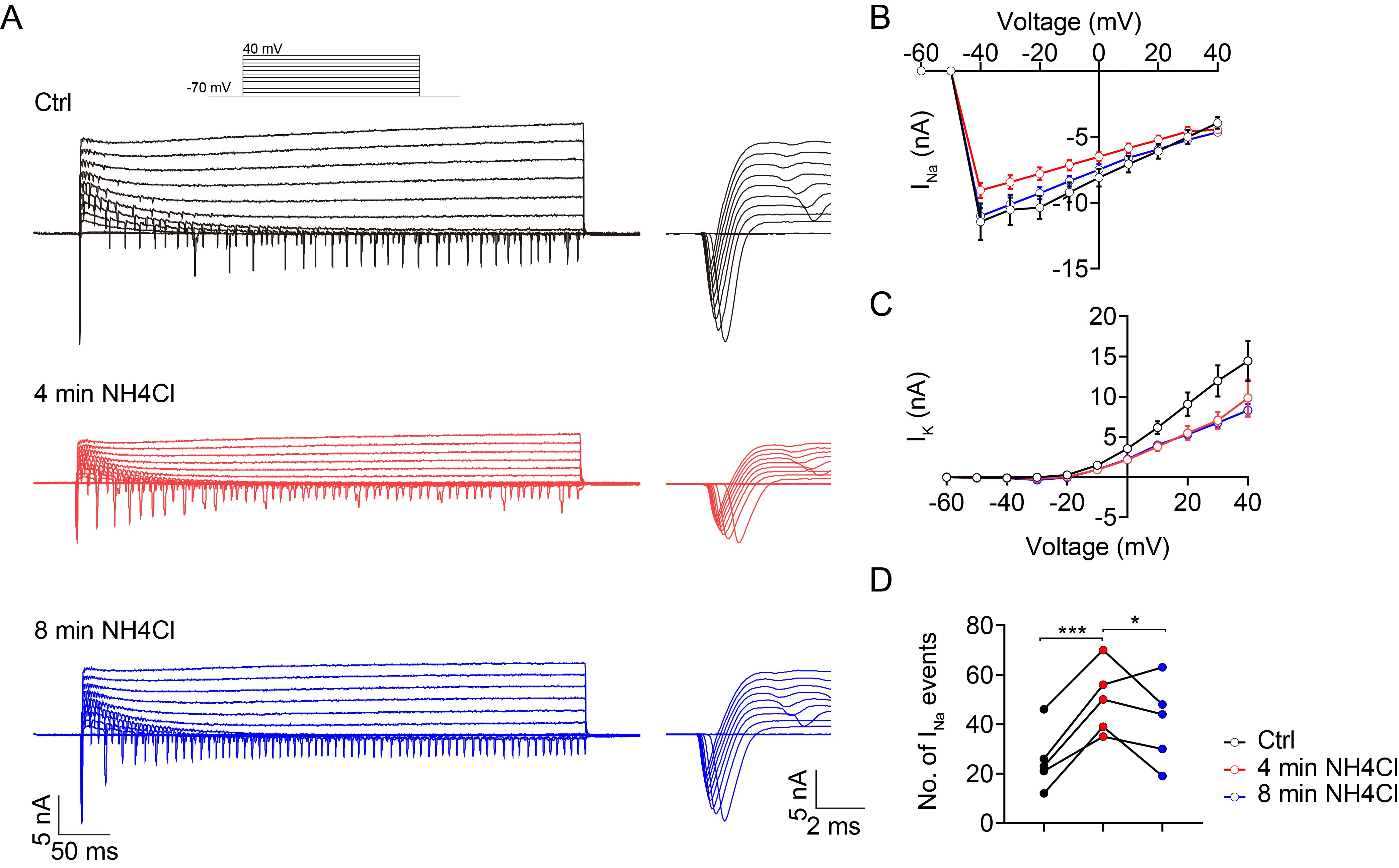

Supplement: Supplementary file 3 — NH4+-induced changes of voltage-gated sodium and potassium currents. (A) Raw electrophysiological traces showing inward currents (Na+ current, INa) and outward currents (K+ current, Ik) in Ctrl, 4-min and 8-min NH4Cl. The protocol was performed by injecting step voltages from −70 mV to 40 mV with the increment of 10 mV at the holding potential of −70 mV in the presence of a cocktail of postsynaptic receptors blockers. Right panel representing the expanded INa in left panel. (B,C) Voltage-current relationship of INa and Ik in Ctrl, 4-min and 8-min NH4Cl. (D) The number of activated INa events when injecting the voltage of −40 mVin Ctrl, 4-min and 8-min NH4Cl. Error bars represent standard error; *p < 0.05, ***p < 0.001; one-way ANOVA with LSD post hoc test. [file Image_3.JPEG]
